# *Pasteurella multocida* Bacteremia in a Patient With Ovarian Cancer and Chemotherapy-induced Neutropenia

**DOI:** 10.1155/S1064744995000639

**Published:** 1995

**Authors:** A. Catherine Casey, Jeffrey S. Greenspoon, Leo D. Lagasse

**Affiliations:** Divisions of Gynecologic Oncology and Maternal-Fetal Medicine Department of Obstetrics and Gynecology Cedars-Sia Medical Center 8700 Beverly Boulevard, 160 West Towers Los Ageles CA 90048 USA

## Abstract

*Background:*  *Pasteurella multocida* is a commensal organism found in the saliva and oropharynx of domestic animals. It causes a variety of human infections ranging from cellulitis to bacteremia and sepsis. The severity of infection is somewhat related to the immunocompetency of the infected host. An immunocompromised host is more likely to suffer a disseminated infection as a result of contact with this organism than an immunocompetent host. This case report and review of the literature are presented to further evaluate the types of infections caused by this organism in oncology patients.

*Case:* A 54-year-old woman with epithelial ovarian cancer and a chemotherapy-induced nadir of her WBC count developed *P. multocida* bacteremia after she incurred a scratch from her pet cat. She was treated with ceftazidime and then penicillin G with prompt resolution of the bacteremia.

*Conclusion:* This paper summarizes an infectious complication that is likely to become more common as chemotherapy-induced neutropenia and pet ownership in the elderly become common coincidences. As such, oncologists and infectious disease physicians should keep this organism in mind when selecting antibiotics to treat the febrile, nadiring cancer patient who has known pet contact.


Infectious Diseases in Obstetrics and Gynecology 3:205–209 (1995)
© 1996 Wiley-Liss, Inc.

### *Pasteurella multocida* Bacteremia in a Patient With Ovarian Cancer and Chemotherapy-Induced Neutropenia

A. Catherine Casey, Jeffrey S. Greenspoon, and Leo D. Lagasse
*Divisions of Gynecologic Oncology and Maternal-Fetal Medicine, Department of Obstetrics and Gynecology, Cedars-Sinai Medical Center, Los Angeles, CA*

ABSTRACT

***Background:* Pasteurella multocida is a commensal organism found in the saliva and oropharynx of domestic animals. It causes a variety of human infections ranging from cellulitis to bacteremia and sepsis. The severity of infection is somewhat related to the immunocompetency of the infected host. An immunocompromised host is more likely to suffer a disseminated infection as a result of contact with this organism than an immunocompetent host. This case report and review of the literature are presented to further evaluate the types of infections caused by this organism in oncology patients.**

***Case:* A 54-year-old woman with epithelial ovarian cancer and a chemotherapy-induced nadir of her WBC count developed *P. multocida* bacteremia after she incurred a scratch from her pet cat. She was treated with ceftazidime and then penicillin G with prompt resolution of the bacteremia.**

***Conclusion:* This paper summarizes an infectious complication that is likely to become more common as chemotherapy-induced neutropenia and pet ownership in the elderly become common coincidences. As such, oncologists and infectious disease physicians should keep this organism in mind when selecting antibiotics to treat the febrile, nadiring cancer patient who has known pet contact.** © 1996 Wiley-Liss, Inc.

KEY WORDS
Immunocompromise, animal bites, malignancy

*Pasteurella multocida* is a gram-negative coccobacillus that exists as a commensal organism in the gastrointestinal and respiratory tracts of domestic animals. It can be isolated from the secretions of 70–90% of cats[1,2] and 50–66% of dogs.[3,4] Human infections most often result from cat bites, cat scratches, or dog bites.[5] However, other routes of infection have been described: direct contamination of a wound with animal saliva;[6] a pulmonary infection from the aerosolized secretions of a cat;[7] pneumonia caused by direct extension from respiratory colonization with this organism in a pet owner;[5] and chorioamnionitis caused by ascending infection from vaginal colonization with this organism in an animal handler.[8]

*P. multocida* may cause a wide spectrum of infections.[5] Soft-tissue infections resulting from animal bites are the most common. Less frequent, but more severe, infections are bone and joint infections;[9] respiratory infections including bronchitis, pneumonia, and empyema;[7] bacteremia and endocarditis;[10–14] central nervous system infections such as meningitis, abscesses, and subdural empyema;[15] subacute bacterial peritonitis;[16] perinatal infections including chorioamnionitis and neonatal sepsis;[6,8,17] and ocular infections ranging from corneal ulcers to endophthalmitis.[5]

Severe infections caused by this organism are more likely to occur in patients who are immunocompromised or have chronic underlying medical

Address correspondence/reprint requests to Dr. A. Catherine Casey, Division of Gynecologic Oncology, Department of Obstetrics and Gynecology, Cedars-Sinai Medical Center, 8700 Beverly Boulevard, 160 West Towers, Los Angeles, CA 90048.

Received October 13, 1995
Accepted December 6, 1995

disorders. The most common chronic medical disorder associated with *P. multocida* infections is liver cirrhosis.[5,12,13,16] *P. multocida* infection has also been reported in association with the following medical conditions: a variety of malignancies, renal failure, systemic lupus erythematosis, chronic lung disease, acquired immunodeficiency syndrome (AIDS), diabetes, paraplegia, rheumatoid arthritis, and the relatively immunocompromised state of pregnancy.[5,6,11,18]

We report a case of *P. multocida* bacteremia in a patient with ovarian cancer who became neutropenic after treatment with chemotherapy. Although a small number of patients with malignancies and *P. multocida* bacteremia have been reported, we are unaware of cases directly associated with ovarian carcinoma or chemotherapy-induced neutropenia.

### CASE REPORT

A 54-year-old white nulligravida was in good health until 1993 when she noted the gradual onset of abdominal distension. A pelvic mass was noted on pelvic examination and confirmed by a pelvic ultrasound. She underwent a total abdominal hysterectomy, bilateral salpingo-oophorectomy, omentectomy, pelvic lymphadenectomy, appendectomy, and optimal debulking. A pathological examination revealed a stage IIIC, grade 2–3, serous cystadenocarcinoma of the ovary. She subsequently received 4 of 6 cycles of cyclophosphamide (500 mg/m$^2$) and carboplatin (350 mg/m$^2$) at 4-week intervals.

Nine days after her 4th cycle of chemotherapy, she developed a severe generalized headache, sore throat, and experienced an episode of emesis. During the night, she experienced rigors with a temperature of 38.3°C. Her malaise worsened the following morning, and she noted a tender left anterior cervical lymph node. She took acetominophen (625 mg) and sought medical care.

She appeared pale and ill. Her temperature was 37.5°C, heart rate 80, blood pressure 100/60, and respiratory rate 20/min. She had a tender, slightly enlarged left anterior cervical lymph node. There was no pharyngeal erythema or exudate. She had a few old cat scratches on her back and a recent noninflamed scratch on her right wrist. Two portacaths present subcutaneously in her upper right chest wall were not inflamed. The remainder of her examination was unremarkable. Her WBC count was 6,200 mm$^3$; the absolute neutrophil count (ANC) was 5,800 including 74% mature neutrophils and 20% band forms. She was admitted to the hospital because of her fever, malaise, and ill appearance. Blood cultures were obtained from her portacaths as well as from a peripheral vein. A throat culture and urine culture were also obtained. Her temperature increased to 39.1°C. Two more sets of blood cultures were obtained and ceftazidime, 2 g q 8 h, was administered. She defervesced rapidly on antibiotics and felt dramatically better 24 h later. Within 24 h, the blood cultures yielded a g-negative coccobacillus which was identified as *P. multocida.* The organism was sensitive to ampicillin, cefazolin, gentamicin, mezlocillin, and ciprofloxacin. The antibiotic therapy was changed to penicillin G, 4 million units q 6 h, after a 3rd set of blood cultures was obtained from each of her portacaths. By the 2nd hospital day, she developed leukopenia with a WBC count of 2,500 mm$^3$ (ANC of 1,575 mm$^3$). Granulocyte Colony Stimulating Factor (G-CSF) was begun. She was discharged home on the 4th hospital day when her 3rd set of blood cultures remained negative at 48 h. She completed 7 days of intravenous (IV) penicillin G and 7 days of oral amoxicillin.

After *P. multocida* was identified, additional information regarding animal contact was elicited from the patient. She had 3 cats and 2 dogs at home. She was advised to declaw the cats and to avoid direct contact with pet saliva during any periods of neutropenia.

### DISCUSSION

*P. multocida* is a microorganism encountered frequently in everyday life, especially among pet owners. In the moderately immunocompromised patient, this encounter can result in a serious and sometimes fatal infection. The medical condition most frequently associated with serious *P. multocida* infections is hepatic cirrhosis. *P. multocida* bacteremia in patients with cirrhosis results in mortality rates of 31% and 37%, as reported by Raffi et al.[11] and Weber et al.,[5] respectively.

Oncology patients also have increased susceptibility to infections with this organism, probably as a result of immunosuppression caused by their malignancies and by chemotherapy and radiation therapy. At least 19 cases of cancer patients with infections caused by *P. multocida* have been reported (Table 1). The reported cases include patients with both hematologic malignancies and various solid

TABLE 1. Clinical features and outcome of oncology patients with *P. multocida* infections[a]

| Case no.[b] | Years of age | Tumor type | Other medical problem | Infection | Antibiotic treatment | Animal exposure | Outcome |
|---|---|---|---|---|---|---|---|
| 1[5] | 69 | Lung carcinoma | COPD | Pneumonia | Cefazolin, tobramycin, cephalexin | Pet dog | R |
| 2[10] | 74 | Bladder-transitional cell carcinoma | | Bacteremia, cellulitis | Gentamicin, ampicillin, penicillin | Cat and dog scratches and bites | R |
| 3[11] | 70 | Macroglobulinemia (CT) | | Bacteremia | Ampicillin, amikacin | Pet dog | R |
| 4[11] | 81 | Chronic lymphocytic leukemia | | Bacteremia, cellulitis | Penicillin, cephalothin, gentamicin | Cat bite | R |
| 5[12] | 18 | Hepatocellular carcinoma | Biliary cirrhosis | Bacteremia | Ampicillin | Pet dog | D |
| 6[13] | 65 | Metastatic breast carcinoma (CT) | | Bacteremia | Penicillin | Pet cat | R |
| 7[15] | 62 | Metastatic squamous cell carcinoma of the tongue (CT, RT) | Alcoholism | Subdural empyema | Ampicillin | Pet dog | R |
| 8[19] | 56 | Rectosigmoid adenocarcinoma | Congestive heart failure | Recurrent bacteremia, perirectal abscess | Cephalothin, gentamicin, clindamycin | Pet dog | D |
| 9[19] | 72 | Biliary adenocarcinoma (RT) | Liver transplant immunosuppression | Recurrent bacteremia, subhepatic abscess | — | — | D |
| 10[19] | 41 | Acute monoblastic leukemia (CT, N) | | Thigh abscess | — | — | — |
| 11[19] | 60 | Metastatic breast adenocarcinoma | | Arm ulcer infection | — | — | — |
| 12[19] | 79 | — | Emphysema | Sputum colonization | — | — | — |
| 13[19] | 60 | Lung carcinoma | Pulmonary fibrosis, bronchiectasis | Sputum colonization | — | — | — |
| 14[19] | 57 | Acute myelogenous leukemia (CT, N) | Pulmonary embolism, systemic aspergillosis | Sputum colonization | — | — | — |
| 15[19] | 75 | Ameloblastoma | | Pneumonia | — | — | — |
| 16[19] | 64 | Metastatic cervical carcinoma | | Vaginal infection | — | — | — |
| 17[19] | 65 | Gingival carcinoma | Congestive heart failure, bronchitis | Sputum colonization | — | — | — |
| 18[19] | 55 | Soft palate epidermoid carcinoma | Cirrhosis | Pneumonia | — | — | — |
| 19[c] | 54 | Ovarian carcinoma (CT, N) | | Bacteremia | Ceftazidime, penicillin G | Cat scratch, pet cats and dogs | R |

[a]CT = chemotherapy; COPD = chronic obstructive pulmonary disease; RT = radiation therapy; N = neutropenia; R = recovered; D = died; — = not available.

[b]Superscript numbers refer to reference citations.

[c]Present study.

tumors. Eight of 19 (42%) of the oncology patients had bacteremia caused by *P. multocida.* Four of these 8 patients also had simultaneous localized infections such as soft-tissue cellulitis. Three of 8 (37%) patients with bacteremia died from their infections. Two of the 3 deaths occurred in patients with underlying liver disease.

Pulmonary infections were the 2nd most common infection, occurring in 7 of 19 (37%) patients; 3 patients had pneumonia and 4 patients had sputum colonized with *P. multocida.* Five of the 7 (71%) patients with pulmonary infections caused by *P. multocida* had associated underlying pulmonary disorders. The other infections reported in oncology patients included abscess formation (N = 3), ulcer infection (N = 1), and a vaginal infection (N = 1).

Nine of the 19 (47%) patients had known animal exposures. Only 3 (33%) of these exposures were traumatic. This rate of traumatic exposure to animals is similar to the 41% reported by Raffi et al.[11] in their review of *P. multocida* sepsis. Thus, other portals of entry of this microorganism may exist in the cancer population, e.g., aerosolized animal secretions, vaginal or pulmonary colonization, or contamination of skin wounds or ulcers with animal saliva.

There are only 3 reported cases of *P. multocida* infections occurring in cancer patients with neutropenia as a result of chemotherapy. Two patients had localized infections (cases 10 and 14, Table 1). The current case report is the only case known to the authors of *P. multocida* bacteremia occurring during a chemotherapy-induced nadir (case 19, Table 1).

*Pasteurella* is usually exceedingly sensitive to antibiotic treatment. The drug of choice is penicillin G, although penicillin-resistant strains have been described.[5,10,11,20] Alternative drugs that are usually effective include ampicillin, carbenicillin, mezlocillin, parenteral cephalosporins, tetracycline, and chloramphenicol. The organism is variably sensitive to the aminoglycosides and erythromycin. *P. multocida* is universally resistant to vancomycin and clindamycin.

Empiric broad-spectrum antibiotic coverage of the febrile and neutropenic cancer patient has been a mainstay of therapy in oncology. Since the 1980s, the number of these patients with documented sites of infection has decreased from 75% to 30–40%, probably attributable to prompt initiation of empiric antibiotic therapy in this setting. Additionally, the types of pathogens predominantly causing infections in this group of patients have shifted from gram-negative bacteria (*Escherichia coli, Klebsiella pneumoniae,* and *Pseudomonas aeruginosa*) to gram-positive organisms (coagulase-negative staphylococci, *Staphylococcus aureus,* and streptococci) as well as fungal, viral, and parasitic pathogens.[21] The initial therapy usually consists of a 3rd-generation cephalosporin, such as ceftazidime, or imipenim alone or an extended-spectrum penicillin with an aminoglycoside or 3rd-generation cephalosporin. All of these empiric regimens would be efficacious in initially treating infections caused by *P. multocida* but should be modified once the antibiotic sensitivities of the organism are known for the reasons previously discussed. Vancomycin is generally reserved for those febrile neutropenic patients in whom a specific pathogen is isolated that justifies its use. Modifications in the initial antibiotic regimen should be made promptly in response to changes in the clinical picture or changes in the microbiologic profile.

The addition of hematopoietic cytokines such as granulocyte (G-) and granulocyte-macrophage (GM−) colony-stimulating factors (CSF) to the treatment regimen of the oncology patient with fever and neutropenia results in a shortening of the duration of absolute neutropenia. Because of the expense of these cytokines, the guidelines for their use made by our institutional pharmacy designate specific groups of patients: patients needing rescue from severe (ANC of <500 or <1,000 and dropping) drug-induced or infection-induced bone marrow failure (with or without fever); patients having received a previous course of the same chemotherapeutic regimen that resulted in profound (ANC of <500) neutropenia or neutropenic infections; and patients undergoing bone-marrow or peripheral stem-cell transplantation. These cytokines are discontinued when the ANC is >2,000 or the WBC is >5,000 following the expected chemotherapy-induced neutrophil nadir.

In summary, *P. multocida* should be considered a potential infectious agent causing bacteremia in oncology patients who are febrile or neutropenic, particularly if they have histories of exposure to domestic animals or underlying medical disorders such as cirrhosis or chronic pulmonary disease. Although the benefits of animal companionship outweigh the risk of infection to the oncology patient with a pet, we feel that physicians should keep in mind the potential animal exposures in this group of patients and readily provide advice for safe practices of pet ownership, especially during periods of chemotherapy-induced neutropenia.

### REFERENCES

1. Owen CR, Buker EO, Be JF, Jellison WJ: *Pasteurella multocida* in animal mouths. Rocky Mount Med J 65:45–46, 1968.
2. Smith JE: Studies on *Pasteurella septica* II. Some cultural and biochemical properties of strains from different host species. J Comp Pathol 68:315, 1958.
3. Bailie WE, Stowe EC, Schmitt AM: Aerobic bacterial flora of oral and nasal fluids of canines with reference to bacteria associated with bites. J Clin Microbiol 7:223–231, 1978.
4. Saphir DA, Carter GR: Gingival flora of the dog with special reference to bacteria associated with bites. J Clin Microbiol 3:344–349, 1976.
5. Weber DJ, Wolfson JS, Swartz MN, Hooper DC: *Pasteurella multocida* infections. Report of 34 cases and review of the literature. Medicine 63:133–154, 1992.
6. Rollof J, Johansson PJH, Holst E: Severe *Pasteurella multocida* infections in pregnant women. Scand J Infect Dis 24:453–456, 1992.
7. Drabick JJ, Gasser RA, Saunders NB, Hadfield TL, Rogers LC, Berg BW, Drabick CJ: *Pasteurella multocida* pneumonia in a man with AIDS and nontraumatic feline exposure. Chest 103:7–11, 1993.
8. Wong GP, Cimolai N, Dimmick JE, Martin TR: *Pasteurella multocida* chorioamnionitis from vaginal transmission. Acta Obstet Gynaecol Scand 71:384–387, 1992.
9. Chevalier X, Martigny J, Avouac B, Larget-Piet B: Report of 4 cases of *Pasteurella multocida* septic arthritis. J Rheumatol 18:1890–1892, 1991.
10. Morris JT, McAllister CK: Bacteremia due to *Pasteurella multocida*. South Med J 85:442–443, 1992.
11. Raffi F, Barrier J, Baron D, Drugeon HB, Nicolas F, Courtieu AL: *Pasteurella multocida* bacteremia: Report of thirteen cases over twelve years and review of the literature. Scand J Infect Dis 19:385–393, 1987.
12. Heyworth MF, Stainforth JN, Wright R, Graham JM: *Pasteurella multocida* septicaemia associated with chronic liver disease. Br Med J 4:733–734, 1975.
13. Grehn M, Muller F, Hany A, Meier P: *Pasteurella multocida* septicemia not associated with primary liver disease. Eur J Clin Microbiol 3:258–260, 1984.
14. Hombal SM, Dincsoy HP: *Pasteurella multocida* endocarditis. Am J Clin Pathol 98:565–568, 1992.
15. Khan MI, Chan R: *Pasteurella multocida* subdural empyema: A case report. Can J Neurol Sci 8:163–165, 1981.
16. Jacobson JA, Miner P, Duffy O: *Pasteurella multocida* bacteremia associated with peritonitis and cirrhosis. Am J Gastroenterol 68:489–491, 1977.
17. Waldor M, Roberts D, Kazanjian P: In utero infection due to *Pasteurella multocida* in the first trimester of pregnancy: Case report and review. Clin Infect Dis 14:497–500, 1992.
18. Caldeira L, Dutschmann L, Carmo G, Abreu J, Sousa G: Fatal *Pasteurella multocida* infection in a systemic lupus erythematosus patient. Infection 21:254, 1993.
19. Stein AA, Fialk MA, Blevins A, Armstrong D: *Pasteurella multocida* septicemia experience at a cancer hospital. JAMA 249:508–509, 1983.
20. Nadler JP, Freedman MS, Berger SA: *Pasteurella multocida* septicemia. NY State J Med 79:1581–1583, 1979.
21. Pizzo PA: Choosing empiric therapy for febrile neutropenic patients: Emerging pathogens and current treatment strategies. J Crit Ill 10:165–168, 1995.
